# Static one-leg standing balance test as a screening tool for low muscle mass in healthy elderly women

**DOI:** 10.1007/s40520-021-01818-x

**Published:** 2021-03-13

**Authors:** Praval Khanal, Lingxiao He, Georgina K. Stebbings, Gladys L. Onambele-Pearson, Hans Degens, Alun G. Williams, Martine Thomis, Christopher I. Morse

**Affiliations:** 1 Department of Sport and Exercise Sciences, Manchester Metropolitan University, Manchester, M15 6BH UK; 2 Department of Movement Sciences, Physical Activity, Sports and Health Research Group, KU Leuven, Leuven, Belgium; 3 Division of Health Science, Manchester Metropolitan University, Manchester, UK; 4 Institute of Sport Science and Innovations, Lithuanian Sports University, Kaunas, Lithuania; 5 Institute of Sport, Exercise and Health, University College London, London, UK

**Keywords:** Pre-sarcopenia, One-leg standing balance, Screening tool, Skeletal muscle phenotypes

## Abstract

**Background:**

Identification of simple screening tools for detecting lower skeletal muscle mass may be beneficial for planning effective interventions in the elderly.

**Aims:**

We aimed to (1) establish a threshold for one-leg standing balance test (OLST) time for low muscle mass, and (2) test the ability of that threshold to assess muscular impairments in a poor balance group.

**Methods:**

Eyes-open OLST (maximum duration 30 s) was performed with right and left legs in 291 women (age 71 ± 6 years). OLST time was calculated as the sum of the OLST time of right and left legs. Fat-free mass (FFM), skeletal muscle mass (SMM), fat mass, biceps brachii and vastus lateralis sizes; handgrip strength (HGS), elbow flexion maximum torque (MVC_EF_) and knee extension maximum torque (MVC_KE_) were measured. Muscle quality was calculated as MVC_KE_/FFM and physical activity was assessed by questionnaire. Low muscle mass was defined as SMM_relative_ of 22.1%, a previously established threshold for pre-sarcopenia.

**Results:**

The OLST threshold time to detect low muscle mass was 55 s (sensitivity: 0.63; specificity: 0.60). The poor balance group (OLST < 55 s) had higher fat mass (3.0%, *p* < 0.001), larger VL thickness (5.1%, *p* = 0.016), and lower HGS (− 10.2%, *p* < 0.001), MVC_EF_ (− 8.2%, *p* = 0.003), MVC_KE_ (− 9.5%, *p* = 0.012), MVC_KE_/FFM (− 11.0%, *p* = 0.004) and physical activity (− 8.0%, *p* = 0.024) compared to the normal balance group. While after adjusting age, the differences exist for HGS, fat mass and VL thickness only.

**Discussion:**

An OLST threshold of 55 s calculated as the summed score from both legs discriminated pre-sarcopenic characteristics among active, community-dwelling older women with limited potential (sensitivity 0.63, specificity 0.60).

**Conclusion:**

OLST, which can be performed easily in community settings without the need for more complex muscle mass measurement, may help identify women at risk of developing sarcopenia.

**Supplementary Information:**

The online version contains supplementary material available at 10.1007/s40520-021-01818-x.

## Introduction

Sarcopenia is characterized by loss of skeletal muscle mass and muscle strength that contributes to a decline in physical performance with ageing [1]. The presence of sarcopenia is dependent on the elderly falling below thresholds of muscle strength and mass to levels rarely seen in those living independently. Skeletal muscle-related impairments such as functional impairment and physical disability may result due to low skeletal muscle mass [2] before reaching these sarcopenic thresholds. Therefore, early identification of skeletal muscle mass below a pre-sarcopenic threshold (i.e., 22.1% skeletal muscle mass relative to body mass, hereafter termed “low muscle mass” [2]), may facilitate appropriate interventions—primarily related to combination of nutrition and exercise [3]–to reverse or slow down the process of muscle wasting and prolong independent living. A simple screening tool that could be conducted with minimal equipment could help to identify those elderly individuals with low muscle mass, who are at risk of sarcopenia and subsequent frailty and loss of independence. One would expect interventions to be more effective at pre-sarcopenic levels of muscle mass than at the accepted thresholds for developed sarcopenia as by then limitations in a physical function may prevent meaningful improvements to the above functional impairment thresholds. Current sarcopenia thresholds for muscle mass are derived with techniques such as magnetic resonance imaging (MRI), bioimpedance analysis (BIA) or dual-energy X-ray absorptiometry (DXA) [1] which may not be universally available. At the most basic functional level, even assessing handgrip strength (a valid measure of the functional aspect of sarcopenia) requires a specialist equipment [4]. An alternative and more accessible screening assessment for low muscle mass in the elderly, that could be adopted in populations living independently is the static one-leg standing balance test (OLST).

Postural stability, an important parameter to explain the state of neuromuscular health, describes an individual’s ability to maintain postural control during a stable position, voluntary movement and reaction to external disturbances without falling [5]. The reduction in dynamic and static balance with ageing is multifaceted, and attributed to both the central nervous and neuromuscular system [6] with fewer receptor cells in vestibular organs [7], alteration in sensory perception and slowing of reaction times [8]. Ultimately, it is the inability of the neuromuscular system to respond to minor postural perturbations that likely result in the association between weak muscles and poor balance with ageing. While several testing procedures for the assessment of balance impairment with ageing exist, the static OLST is easy to adopt in clinical and geriatric settings to assess postural steadiness in a static position [9]. Although there is inconsistency in the use of single leg or summed times, maximum duration of tests, and eye condition (opened or closed) among existing studies [10], the OLST has been successfully associated with muscle strength and frailty [11, 12]. To date, the OLST has been adopted as a screening tool for balance impairment [10], whereby failing to pass a 5 s threshold has been associated with poor functional status [13] and impaired ability to perform activities of daily living (ADLs) [14]. Importantly, it should be noted that this 5 s threshold has been derived mostly in either very weak and frail populations or older populations [13–15]. Falls and fall-related injuries are the primary cause of hospitalization among the elderly and signal the initial stage of loss of independence [16] and show an association with both lower skeletal muscle mass [17] and poor balance [18]. Therefore, identification of an OLST-time threshold for pre-sarcopenic risk in active community dwellers may allow an alternative screening method to evaluate musculoskeletal health and via subsequent interventions reduce the risk of adverse outcomes such as injurious falls associated with low muscle mass.

While the 5 s threshold is associated with poor functional status in weak and frail populations, this threshold is unable to detect low muscle mass because active, independently living older people will exceed this threshold easily [19]. Furthermore, since there can be significant differences in OLST time between left and right legs [20], a summed score of both legs in a balance test maybe more appropriate for identifying the threshold risk of low muscle mass. Thus, we reasoned that for the detection of low muscle mass a different OLST threshold is needed that could be recommended in clinical and geriatric settings if associated with other clinically relevant pre-sarcopenic characteristics in older individuals with poor balance. Therefore, the aim of the present study was to (1) identify an OLST time threshold for pre-sarcopenic risk of low muscle mass, (2) test how the identified OLST time threshold is associated with the pre-sarcopenic characteristics body composition, regional muscle size, muscle strength, muscle quality and physical activity level.

## Materials and methods

### Participant characteristics and inclusion criteria

Older women (*n* = 291, 60–91 years, age 71 ± 6 years) were mainly recruited via the University of the Third Age, Cheshire region, UK, or via word-of-mouth. All participants were 60 + years, self-reported as being free from any issues that affected their daily activities and physical independence. Individuals were excluded if they had a history of neuromuscular or cardiovascular conditions, including a self-reported history of vertigo.

### One-leg standing balance test

The one-leg standing balance test (OLST) was performed with both the right and left leg alternately. Participants were asked to stand unshod and then to stand on one leg, whilst flexing the contralateral knee at 90° behind them, whilst maintaining a vertical thigh position, parallel to the standing leg. The test was performed alternately with the right and left leg with 10 s rest between trials. If they completed 30 s of one-leg standing (recorded with stopwatch), then it was recorded as OLST for that specific leg [21]. If they did not achieve 30 s, they were allowed a maximum of three attempts until they achieved 30 s, or when they failed to achieve 30 s the maximum time among the three attempts was recorded as their specific leg OLST. OLST total was calculated as the sum of OLST time of the right OLST (RL) and left leg OLST (LL): OLST = OLST (RL) + OLST (LL). Test-re-test reliability is moderate (ICC = 0.60) [22] to high (ICC > 0.90) [23] for the OLST.

### Body composition and muscle size

Skeletal muscle mass (SMM) and fat mass were estimated by bio-impedance analysis (Model 1500; Bodystat, Isle of Man, UK) as described in our previous work [24].

During the procedure, participants lay in a supine position for 5 min on a physiotherapist bed to ensure homogeneous distribution of body fluid, followed by attaching the adhesive electrodes to the dorsum of the right hand and leg. Subsequently, a small current was passed between the attached electrodes.

Skeletal muscle mass was estimated using an equation previously validated in a Caucasian population [25] as:

Skeletal muscle mass (SMM)=Ht^2^/(*R* × 0.401)−0.071 × age + 5.102,

Where, Ht is height of the individual in m, *R* is resistance from the device in Ohm and age is in years. The SMM has shown a high correlation with DXA measures [26]. Subsequently, fat-free mass (FFM) was calculated from the recorded fat mass as body mass—fat mass.

An ultrasound (MyLab^™^Twice, Esaote Biomedical, Italy) was used to perform the scan to measure the biceps brachii thickness at 60% length from the proximal end of the humerus [27, 28].

For vastus lateralis (VL) thickness, the origin and insertion of the VL were identified and sagittal scans at 50% VL length were performed. The mean of three thicknesses measured at proximal, middle and distal points between superficial and deep aponeuroses was recorded as VL thickness.

### Muscle strength and quality

Hand grip strength (HGS) was measured with a dynamometer (JAMAR plus, JLW Instruments, Chicago, USA) as described in our previous work [27].

Similarly, maximum isometric elbow flexion torque (MVC_EF_) and knee extension torque (MVC_KE_) were measured with dynamometer with a load cell (Zemic, Eten-Leur, Netherlands). The detailed procedure has been described in our previous work [27].

Lower limb muscle quality was defined as the knee strength relative to fat-free mass (MVC_KE_/FFM) [29].

### Physical activity

The PASE (Physical activity scale for elderly) questionnaire was used to evaluate physical activity level [30].

### Assessment of low muscle mass

Low muscle mass was defined as having a relative skeletal muscle mass (SMM_r_) < 22.1% [2], calculated as 100 × SMM/Body mass. Based on this threshold for low muscle mass, the corresponding OLST threshold was calculated (see below).

### Statistical analysis

Statistical analyses were carried out using SPSS Version 26.0 for Windows (IBM Corp., Armonk, NY, USA) and *p* < 0.05 was considered statistically significant. Throughout, low muscle mass was defined as individuals with SMM_r_ < 22.1% [2]. Kruskal–Wallis tests were performed for continuous variables for the group-wise comparisons with Bonferroni correction to adjust for multiple comparisons. Spearman correlations were performed to identify the strength of association of OLST with %SMM, age and BMI; and BMI with muscle-related phenotypes. Variables significantly correlated were used in multivariable linear regression analysis to predict the variance of the OLST. To establish the threshold value of OLST that predicted pre-sarcopenic risk, receiver operating characteristic (ROC) curve analysis was performed. OLST time threshold was defined as that with the highest values of sensitivity and specificity for distinguishing between low muscle mass and healthy muscle mass participants. The area under the ROC curve was used to assess the discriminatory ability of the model. Based on the ROC analysis, participants were then divided into those of “poor” (summed OLST < 55 s, see below) and “normal” (summed OLST ≥ 55 s) balance. Kolmogorov–Smirnov tests were completed to check for normal distribution of neuromuscular phenotypes in the poor balance and normal balance groups. For phenotypes meeting parametric assumptions (with both poor balance and normal balance groups), between-group analyses were conducted using independent samples t tests. For phenotypes that violated parametric assumptions, Mann–Whitney tests were used and Monte–Carlo *p* values are reported. Further to these, analysis of covariance (ANCOVA) was also used to investigate if the differences remained after using age as covariate. Unless stated otherwise, parametric data are presented as mean ± SD, with non-parametric data presented as median (inter-quartile range).

## Results

### Distribution of participants with low muscle mass prevalence and balance performance

**Fig. 1:**
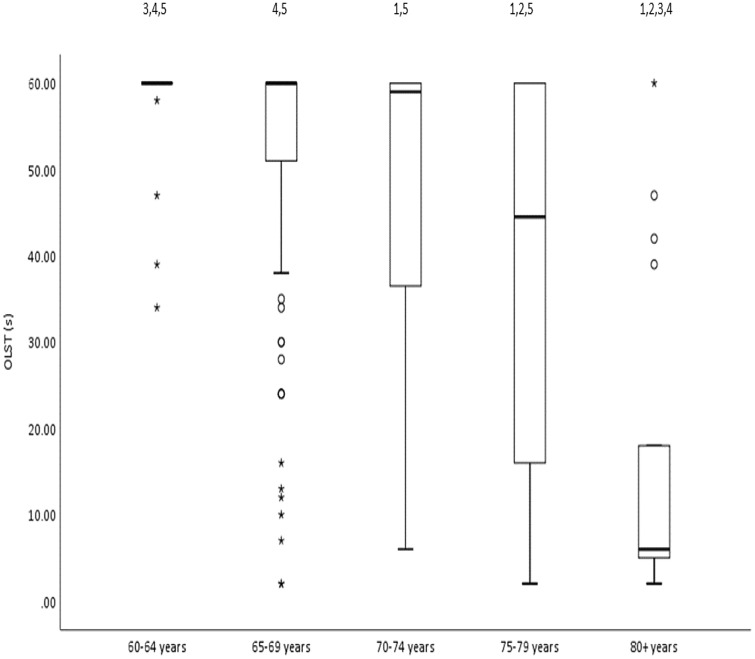
Boxplot showing OLST time for five 5-year age groups. Circles and asterisks indicate outliers and extreme outliers, respectively. *OLST* one-leg standing balance test. ^1,2,3,4,5^ show difference from 60–64 years, 65–69 years, 70–74 years, 75–79 years and 80 + years, respectively (Kruskal–Wallis test with Bonferroni correction for multiple comparisons) at *p* < 0.05

Fifty-five percent (*n* = 161) of the participants passed the 30 s OLST with both right and left legs, with older groups showing a lower 60 s pass prevalence than younger groups (Table 1). OLST time was lower in older age groups, such that the 80 + years group only achieved 29% of OLST time achieved by the 60–64 years group (Fig. 1). Only one participant was identified as sarcopenic (having both lower skeletal muscle mass (%SMM_r_ < 22.1%) and lower HGS (HGS < 19 kg)) in the current older population. The prevalence of % SMM_r_ low muscle mass increased (though unevenly) with age from 8% in the 60–64 year group to 29% in the 80 + years group (Table 1). Most participants (*n* = 261, 90%) exceeded 5 s OLST on both legs.

**Table 1:** Distribution of participants across age groups and their categorizations according to body mass index, % skeletal muscle mass, achieving maximum 60 s one-leg standing balance test and low muscle mass prevalence

Age-group (years)	Number of participants	BMI (kg/m^2^)	% SMM_r_	% individuals failing to achieve summed 60 s OLST	Low muscle mass prevalence using % SMM_r_ [%(*n*)]
60–64	40	25.2 ± 4.0	27.2 ± 3.7	10.0%	7.5% (3)
65–69	104	26.3 ± 4.9	25.7 ± 3.7	36.5%	16.3% (17)
70–74	96	25.8 ± 3.9	25.4 ± 4.3	51.0%	13.5% (13)
75–79	34	25.1 ± 3.3	26.0 ± 3.3	67.6%	5.9% (2)
80 +	17	28.4 ± 3.4	23.6 ± 3.1	94.1%	29.4% (5)
All participants	*n* = 291	25.9 ± 4.2	25.8 ± 3.9	45.0%	13.7% (40)

*BMI* body mass index, *OLST* one-leg standing balance test *SMM* skeletal muscle mass. Data are presented as mean ± SD for BMI and %SMM_r_

### Association of % SMM_r_, OLST, body composition and muscle related phenotypes

OLST time was moderately and positively correlated with % SMM_r_ (*ρ* = 0.352, *p* < 0.001) and moderately and negatively with BMI (*ρ* = **−** 0.320, *p* < 0.001) and age (*ρ* = **−** 0.446, *p* < 0.001). Similarly, BMI was moderately and positively correlated with biceps brachii thickness (*ρ* = 0.226, *p* < 0.001) and VL-thickness (*ρ* = 0.311, *p* < 0.001), strongly and positively correlated with fat mass (*ρ* = 917, *p* < 0.001), and moderately and negatively correlated with MVC_KE_/FFM (*ρ* = **−** 0.236, *p* < 0.001) while it was not correlated with HGS (*ρ* = 0.037, *p* = 0.526), MVC_EF_ (*ρ* = 0.111, *p* = 0.058), MVC_KE_ (*ρ* = 0.011, *p* = 0.853) and physical activity (*ρ* = **−** 0.079, *p* = 0.181). Multivariable linear regression with BMI and %SMM_r_ included in the model as predictors predicted approximately 14% of the variance in OLST time.

**Fig. 2:**
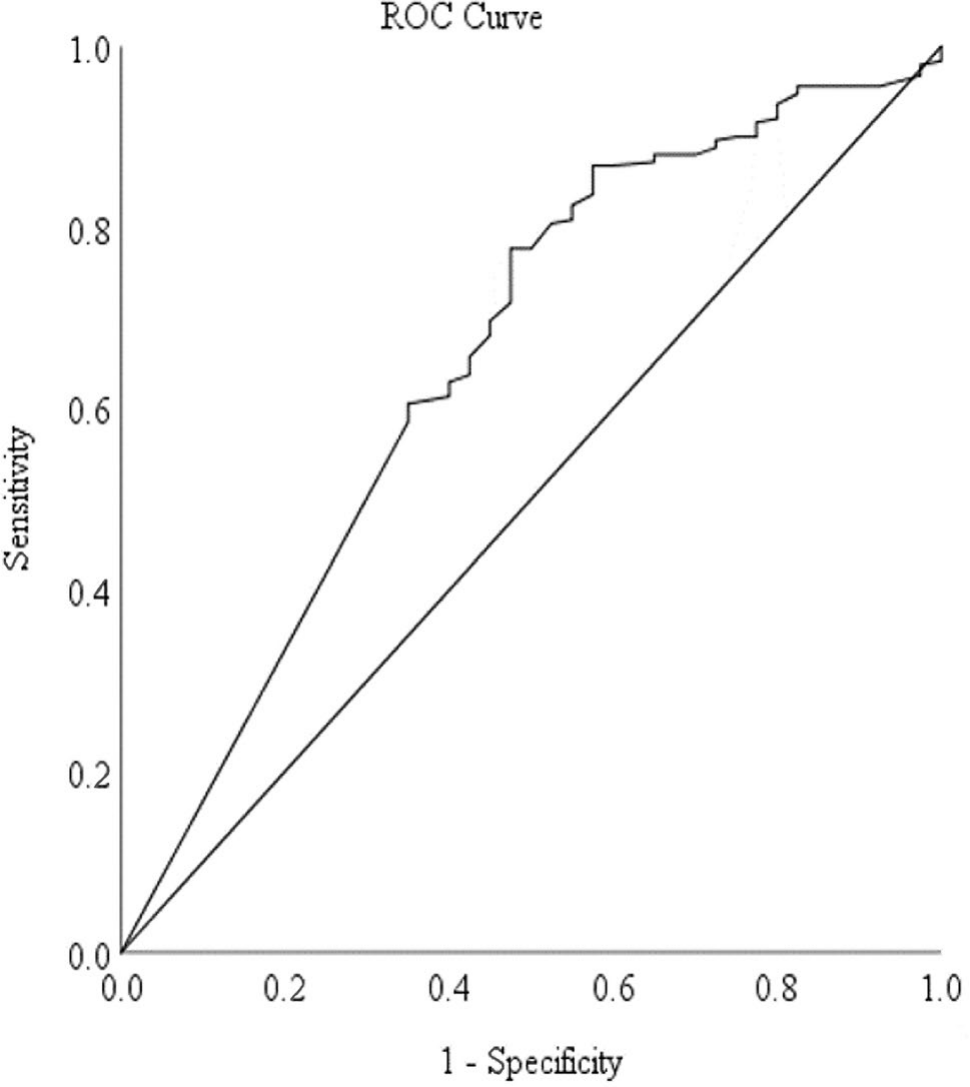
ROC analysis for OLST time threshold based on low muscle mass threshold

Upon ROC analysis that predicted the low muscle mass threshold of 22.1% SMM_r_, the AUC for the predicted model was 0.65 (Fig. 2). The threshold for OLST time was 55 s that corresponded to the highest sensitivity and specificity values (sensitivity 0.63; specificity 0.60).

### The OLST threshold and low muscle mass features

The prevalence of poor balance (below 55 s OLST) was 40% (*n* = 117). Compared to the normal balance group, the poor balance group showed lower HGS (**−** 10.2%, *p* < 0.001), MVC_EF_ (**−** 8.3%, *p* = 0.008), MVC_KE_ (**−** 9.5%, *p* = 0.012), MVC_KE_/FFM (**−** 11.0%, *p* = 0.004) and physical activity level (**−** 8.0%, *p* = 0.024) (Table 2). Similarly, there was a higher fat mass (3.0%, *p* < 0.001) and larger VL thickness (5.1%, *p* = 0.016) in the poor balance group, but no differences in biceps brachii thickness (*p* = 0.325) (Table 2). The poor balance group was older and heavier than the normal balance group (Table 2, *p* < 0.05).

**Table 2:** Participant characteristics of poor and normal balance groups based on the identified threshold of 55 s

Phenotypes	General characteristics	Standing balance test categories	
	(*n* = 291)	Poor balance (*n* = 117)	Normal balance (*n* = 174)
Age (years)	70.6(5.7)	72.7(7.7) **	68.8(5.7)
Body mass (kg)	65.5(13.9)	67.8(14.2) **	63.6(10.9)
Height (m)	1.60 ± 0.06	1.59 ± 0.06	1.60 ± 0.05
BMI (kg/m^2^)	25.3(4.6)	26.4(6.2) **	24.6(4.5)
FFM (kg)	37.8 ± 5.0	38.0 ± 5.2	37.7 ± 4.9
Fat (kg)	27.1(8.8)	29.1(10.6) **	25.7(7.8)
Fat %	42.5 ± 5.1	44.9 ± 4.9 *	40.9 ± 4.6
SMM (kg)	16.8 ± 2.3	16.6 ± 2.2	17.0 ± 2.4
% SMM_r_	25.7 ± 3.9	24.3 ± 3.3 *	26.8 ± 3.9
Biceps brachii thickness (cm)	1.72(0.44)	1.69(0.48)	1.73(0.42)
VL thickness (cm)	1.91 ± 0.35	1.97 ± 0.36 *	1.87 ± 0.34
HGS (kg)	30.1 ± 4.9	28.1 ± 4.7 **	31.3 ± 4.7
MVC_EF_ (N·m)	24.5(8.0)	23.2(7.5) *	25.0(8.0)
MVC_KE_ (N·m)	55.7 ± 18.5	52.4 ± 18.1 *	57.9 ± 18.5
Physical activity	157 ± 50	141(65) *	160(60)
MVC_KE_/FFM (N·m/kg)	1.48 ± 0.48	1.38 ± 0.43 *	1.55 ± 0.48

*BMI* Body mass index, *FFM* fat-free mass, *SMM* skeletal muscle mass, *VL* vastus lateralis, *HGS* handgrip strength, *MVC*_*EF*_ isometric elbow flexion, *MVC*_*KE*_ isometric knee extension. Parametric data are presented as mean ± SD and non-parametric as median (inter-quartile range). * and ** denote *p* < 0.05 and *p* < 0.001, respectively

With age used as covariate, the differences remain between the poor and normal balance groups for HGS (*F* (1, 288) = 11.3, *p* = 0.001), VL-thickness (*F* (1, 288) = 13.8, *p* < 0.001) and fat mass (*F* (1, 288) = 35.4, *p* < 0.001) but not with MVC_EF_ (*F* (1, 288) = 2.4, *p* = 0.121), MVC_KE_ (*F* (1, 287) = 0.4, *p* = 0.512), physical activity level (*F* (1, 287) = 3.4, *p* = 0.067) and MVC_KE_/FFM (*F* (1, 288) = 2.245, *p* = 0.135).

## Discussion

The current study identified an OLST time threshold for low muscle mass risk in healthy elderly women and then tested the potential of this derived OLST threshold to distinguish pre-sarcopenic characteristics in the poor balance group. We identified a low muscle mass OLST threshold of 55 s (OLST = OLST (RL) + OLST (LL); 30 s maximum duration for each leg) in healthy community-dwelling women. The derived 55 s OLST time threshold successfully defined a poor balance group with greater fat mass and lower muscle strength (HGS). We, therefore, suggest that the static one-leg standing balance test could be an accessible alternative to detect presence of low muscle mass in community-dwelling healthy elderly women.

Low-muscle mass is the first stage of sarcopenia detection [1] and is linked to adverse outcome measures such as physical dependence [2] and increased risk of falls [17]. However, the assessment of skeletal muscle mass requires equipment such as MRI, BIA and DXA, which may not be readily available and certainly lack the flexibility to screen for the presence of low muscle mass within a community setting in independently living elderly women. Several alternatives have previously been suggested for the detection of low muscle mass and sarcopenia. For instance, threshold measures of muscle thickness (sensitivity 0.74; specificity 0.17) [31], hand grip strength (sensitivity 0.61; specificity 0.60) [32] and anthropometric indicators such as calf circumference (sensitivity 0.6; specificity 1.0) [31] have been proposed as thresholds in sarcopenia identification. Indeed, HGS is the initial screening method for sarcopenia in elderly women based on EWGSOP2 guidelines [4], but requires a handgrip dynamometer. In contrast, OLST as in the present study requires no more than a timer and can be performed easily and reliably by the participant and investigator [10]. Although the risk of falls during the test should not be ignored, a 5 s threshold in even the frailest elderly is a testament to the safety of the approach.

The 22.1% SMM_r_ threshold for the presence of low muscle mass used in the present study has been previously used to investigate genetic variants associated with sarcopenia [24], and identifying risk of disability [2]. In the present study, OLST was moderately and negatively associated with both age and BMI, while moderately and positively with %SMM_r._ The decrement in OLST time with ageing has been reported before [21] and the positive association of %SMM_r_ with OLST time observed in the present study is in line with other studies showing a better physical performance with higher muscle mass [33, 34]. In the present study, establishing a low muscle mass threshold for OLST was based on ROC analysis using the 22.1% SMM_r_ threshold. We observed an acceptable model prediction (AUC = 0.65) [35] between summed OLST and the 22.1% SMM_r_ pre-sarcopenic threshold, and identified 55 s OLST time as a threshold with slightly lower sensitivity (0.63) and specificity (0.60). The lower AUC of the model and lower specificity and sensitivity of the OLST time threshold identified may be explained by the confounding impact of BMI on OLST time (*ρ* = **−** 0.320, *p* < 0.001) and the positive correlation between %SMM_r_ and OLST time (*ρ* = 0.352, *p* < 0.001). In addition, we should note that %SMM_r_ and BMI combined, predicted 14% of the variance observed for OLST. The negative relation between BMI and OLST observation corresponds with a previous association of higher BMI with lower postural control and balance ability [36]. Thus, BMI may act as confounding factor affecting the physiological parameters of the balance and control in addition to reducing %SMM_r_ (as increment in BMI increases the body mass in denominator while calculating %SMM_r_). Previous studies have shown a positive association of physical performance measures with muscle mass and negative associations with obesity-related indices [37, 38]. One should realise, however, that there is most likely a threshold muscle mass below which OLST may also become limited by the low muscle mass. An upper limit is essential for maintaining the practicality of the test, so future studies could investigate alternatives. However, we should also note that the observed low AUC, sensitivity and specificity may be attributed to other factors related to the nervous system, alteration in sensory perception and slowness in reaction times that are associated with ageing [6–8].The 55 s OLST threshold of low muscle mass in the present study should be considered alongside existing OLST thresholds. A 5 s OLST threshold is established in frail and clinical populations [10, 13, 15] and 9 s for locomotive dysfunction in a Japanese population [39]. In all these instances the lower OLST thresholds are from frail (5 s, [10, 13, 15]) and older participants (9 s [39]). For instance, the present study included younger participants (71 ± 6 vs 77 ± 6 years [39]) scored from both legs [summed score (OLST (RL) and OLST (LL)] time vs one leg OLST time) and included independently living participants, in contrast to those with locomotive dysfunction previously [39]. The derived 55 s OLST is therefore, more applicable for healthy older women who do not have other comorbidities and would ordinarily slip under the radar for dedicated interventions for sarcopenia prevention.

The secondary aim of the current study was to evaluate if the derived 55 s OLST threshold is a suitable pre-screening tool for muscle function, muscle mass and physical activity that have known implications for independence in the elderly. Participants with an OLST below the 55 s threshold had a higher fat mass, larger VL and lower muscle strength (HGS) than their normal-balance counterparts. The lower HGS and higher fat mass observed in the poor balance group has been previously associated with lower skeletal muscle mass [40, 41], and poor scores in functional, psychological and social health domains [42]. The larger VL muscle thickness in the poor balance group in the present study is consistent with greater muscle mass in overweight individuals [43] (high BMI observed in poor balance group in the present elderly women). Furthermore, note that muscle thickness is not the same as muscle quantity and ignores intramuscular fat deposition in obese individuals [44]. The ability to characterize pre-sarcopenic features in our poor balance group suggests the 55 s OLST threshold has potential to screen for pre-sarcopenia in healthy community-dwelling women.

Although the 55 s OLST threshold was somewhat successful in characterizing pre-sarcopenic characteristics, the authors suggest its practical use should be cautious considering the observed sensitivity, specificity and AUC values. The sensitivity and specificity may improve if the test was lengthened to 60 s for each leg (summed 120 s), as 55% participants completed the summed 60 s. We also suggest that thresholds could be population-specific and could be different if alternative indices for low muscle mass are used. We also like to acknowledge that our sample represents a likely healthy bias of participants, and a volunteer bias for those active elderly as all such investigations into health and activity of elderly. Although we adopted an inclusive approach to recruitment, for ethical reasons and participant safety, participants who had cardiovascular, or neuromuscular conditions were excluded. Despite these limitations, understanding muscle atrophy at the initial, stage prior to sarcopenia, via cautious use of the simple OLST test might be useful for designing and planning appropriate interventions such as nutrition and exercise.

The current study in actively living healthy older women provides novel evidence for the potential of using the one-leg standing balance test in the assessment of low skeletal muscle mass and suggests 55 s (summed score) as the optimal threshold for detection. We suggest more cross sectional and longitudinal studies to evaluate the derived OLST threshold’s ability to identify low muscle mass individuals and explore its potential to investigate other possible factors that are associated with low muscle mass in healthy community dwellers. By identifying lower muscle mass using the OLST, earlier personalised targeting of interventions among the elderly may be possible.

## Data availability

The datasets generated during and/or analysed during the current study are available from the corresponding author on reasonable request.

## Supplementary Information

Below is the link to the electronic supplementary material.

Supplementary file1 (XLSX 61 KB)
